# Effect of Dupilumab in CRSwNP Sinonasal Outcomes from Real Life Studies: A Systematic Review with Meta-analysis

**DOI:** 10.1007/s11882-025-01192-y

**Published:** 2025-02-05

**Authors:** Miguel Rodriguez-Iglesias, Christian Calvo-Henríquez, Daniel Martin-Jimenez, Ainhoa García-Lliberós, Juan Maza-Solano, Ramon Moreno-Luna, Adriana Izquierdo-Domínguez, Gabriel Martínez-Capoccioni, Isam Alobid

**Affiliations:** 1 Rhinology group of the Young-Otolaryngologists of the International Federations of Oto-rhino-laryngological Societies (YO-IFOS) study group, Dubai, United Arab Emirates; 2 Service of Otolaryngology, Hospital Complex of Santiago de Compostela, Santiago de Compostela, Spain; 3 Translational Research In Airway Diseases Group (TRIAD), Instituto de Investigación Sanitaria de Santiago de Compostela (IDIS), Santiago de Compostela, Spain; 4 Rhinology and Skull Base Surgery Unit, Department of Otolaryngology, University Hospital Virgen Macarena, Seville, Spain; 5 Department of Otolaryngology, Valencia General University Hospital, Valencia, Spain; 6 Department of Surgery, University of Seville, Seville, Spain; 7 Rhinology and skull base unit. Service of Otolaryngology, Hospital Clinic. IDIBAPS, CIBERES, Universitat de Barcelona, Barcelona, Spain; 8 Department of Allergology, University Hospital of Terrassa, Barcelona, Spain; 9 Alergo-Rino Unit. Tekno medical centre, Barcelona, Spain

**Keywords:** Biologic therapy, Chronic rhinosinusitis with nasal polyps, Dupilumab, Monoclonal antibody, Quality of life

## Abstract

**Purpose of Review:**

Chronic rhinosinusitis with nasal polyps (CRSwNP) is a debilitating inflammatory condition that significantly impacts quality of life. Despite treatment advances, recurrence is common, prompting the exploration of novel therapies such as monoclonal antibodies targeting the type 2 immune response, notably dupilumab. This research aims to evaluate the real-world evidence (RWE) of dupilumab in treating severe CRSwNP, comparing sinonasal outcomes to those observed in randomized clinical trials.

**Recent Findings:**

Significant improvements were noted, with the average SNOT-22 score reduction being 37.2 points post-dupilumab treatment. The nasal polyp size (NPS) showed an average decrease of 3.6 points. The analysis highlighted the practical effectiveness of dupilumab, emphasizing its benefit over conventional therapies in reducing NPS and improving nasal symptoms.

**Summary:**

The findings advocate for the integration of dupilumab into standard treatment protocols for severe CRSwNP, providing a robust alternative that could potentially reduce the high recurrence rates associated with current management strategies. This study underscores the utility of RWE in assessing the effectiveness of new medical treatments, suggesting that dupilumab offers substantial real-world benefits for patients suffering from this challenging condition.

**Supplementary Information:**

The online version contains supplementary material available at 10.1007/s11882-025-01192-y.

## Introduction

Chronic rhinosinusitis with nasal polyps (CRSwNP) represents a specific phenotype of chronic rhinosinusitis implying the development of inflammatory polypoid outgrowths from the nasal mucosa. It is a prevalent and debilitating disease with significant implications for public health and is estimated to affect approximately 4.2% of the general population in the United States, with approximately 0.027% facing severe uncontrolled CRSwNP [1–3]. Some recent studies have reported prevalences < 1% in Catalonia [4] and Spain [5]. Beyond its prevalence, CRSwNP exerts a substantial burden on health-related quality of life [6], affecting various aspects of quality of life (QoL), such as general health, social functioning, sleep and mental health [7], often resulting in absenteeism at work [8]. 

Despite standard treatment combining topical intranasal corticosteroids (INCS)), systemic corticosteroids (SCS), and/or surgical interventions in the presence of severe symptoms, the recurrence rates still remain high, ranging from 40 to 80% within 3 to 12 years after surgery [9–11]. 

In recent years, advances in understanding the underlying inflammatory processes have paved the way for new precision medicine treatments, aimed at controlling the inflammatory cascade [12]. In approximately 80% of Caucasian patients with CRSwNP the disease is caused by type-2 inflammation [4, 13, 14]. The emergence of monoclonal antibodies (mAbs) targeting the type 2 immune response has revolutionized the treatment landscape for conditions associated with type 2 inflammation, including CRSwNP. Currently, the approved mAbs for severe CRSwNP are omalizumab (anti-IgE), dupilumab (anti-IL-4Rα), and mepolizumab (anti-IL-5). Dupilumab is a fully human immunoglobulin G_4_ subclass monoclonal antibody that blocks IL-4 and IL-13 signaling by specifically binding to the IL-4Rα receptor subunit. Thus, it modulates cell function, cell signaling through several chemokines, and immunoglobulin E synthesis [15]. 

In various network meta-analyses [16–18], dupilumab has shown better effects in CRSwNP compared to other mAbs. However, these studies solely focused on comparing randomized clinical trials (RCTs), leading to certain limitations. Variations in study designs, specific characteristics of the study population and biases among trials are among these limitations. Furthermore, the controlled environments of RCTs may hinder the applicability of findings to real-world scenarios (RWE, Real World Evidence), and the short follow-up durations may restrict the assessment of long-term outcomes and safety profiles. Thus, these conditions could raise doubts about the robustness of conclusions. RWE could be an indirect way of comparing results reflecting the conditions and outcomes occurring outside the controlled environment of clinical trials [19] and taking into account that the criteria to prescribe mAbs for CRSwNP are common to all biologics (EPOS2020 [20], POLINA [21], EPOS/EUFOREA update [22]).

The main goal of this research is to summarize in a systematic review with meta-analysis the available evidence on RWE of dupilumab in CRSwNP, and to compare the results obtained from RCTs, providing information on the possible advantages and drawbacks of employing these therapies in our daily clinical routines.

## Methods

### Systematic Review

The review was carried out according to the PRISMA and AMSTAR-2 guidelines. The PROSPERO protocol was published according to the NHS International Prospective Register of Systematic Reviews (Registration No. 541594).

### Literature Search. Inclusion and Exclusion Criteria

The criteria for considering studies for the systematic review were based on the population, intervention, comparison, and outcome (PICOTS) framework.

#### Participants

severe uncontrolled CRSwNP patients.

#### Intervention

dupilumab 300 mg subcutaneously every two weeks.

#### Comparison

pre-and posttreatment data.

#### Outcomes

SinoNasal Outcome Test (SNOT-22), Nasal Polyp Score (NPS).

#### Timing and Settings

included studies were published between 2022 and 2024.

#### Types of Studies

prospective and retrospective studies published in peer-reviewed journals. Case reports and theses were not included. There were no restrictions by date or publication type, and the search was last updated in June 2024. Studies published in languages other than English, Spanish, Italian or Portuguese were excluded.

#### Exclusion Criteria

Different studies were excluded such conference abstracts [23–35], clinical trials or studies consisting of post hoc analysis of clinical trials [36–67], studies in which dupilumab was indicated for other comorbidity different from CRSwNP [68–75], studies with population already included in other studies (duplicated) [76–80], studies in which SNOT-22 or NPS data were not used or were published incomplete [81–93], studies where patients did not meet the inclusion criteria [94–108] and studies published in languages different from english, spanish, portuguese or italian [109–113]. 

### Search Strategy

Five databases were explored: PubMed (Medline), EMBASE, Web Of Science, SciELO, and Trip Database. The search strategy, adapted to the syntax of each database was ((snot-22[Title/Abstract]) OR (“sinonasal outcome test“[Title/Abstract]) OR (nps[Title/Abstract]) OR (“nasal polyp score“[Title/Abstract])) AND ((dupilumab[Title/Abstract]) OR (dupixent[Title/Abstract])).

The abstracts were reviewed by two authors of the Rhinology Study Group of Young Otolaryngologists of the International Federation of Otorhinolaryngological Societies (MRI, CCH), and those that potentially met the inclusion criteria were read in full text. When differences in eligibility judgment were noted, full texts were included for the final assessment. Furthermore, the reference lists of all selected articles were manually reviewed to identify any work that may have been overlooked during the initial search.

### Study Extraction and Analysis

Three authors (MRI, CCH, AGLL) analyzed and extracted data, including sample size, sex, age, type of study, comorbidities (i.e., asthma, NSAID-exacerbated respiratory disease [N-ERD], prior Endoscopic Sinus Surgery [ESS]) and main outcome variables (SNOT-22 [114] and/or NPS [115]). When data were only partially published, common variances were calculated using the formula (√(𝜎_x_^2^ + 𝜎_𝑦_^2^) / √(n_x_ + n_𝑦_)) * 1.96. When the main data were published expressed in median and interquartile range, the mean and standard deviation were estimated using the Wan’s method [116]. 

Follow-up was expressed in weeks. Data were converted assuming 1 month equaled to 4.3 weeks, and 1 year equaled to 52 weeks.

### Statistical Analysis

All statistical data were analyzed using STATA for Macintosh v. 15.1 (StataCorp ^®^). Significance was considered at a *P*-value < 0.05.

Meta-analysis was conducted using rBiostatistic Web Tool (https://www.rbiostatistics.com/one_group_means). Heterogeneity among the included studies was rigorously evaluated through two established tests: the Q-test and the I^2^ test. The Q-test assesses whether the observed variability in effect sizes across studies exceeds what would be expected by chance alone, while the I2 test quantifies the proportion of total variation attributable to heterogeneity rather than random error.

To determine the appropriate statistical model for combining study findings, the level of heterogeneity was pivotal. A fixed-effects model, predicated on the assumption of a common effect size across all studies, was employed when heterogeneity was below 50% and did not exhibit statistical significance (*p* ≥ 0.05). Conversely, a random-effects model, accommodating both within-study and between-study variability, was applied when heterogeneity surpassed 50% or when the *p*-value was < 0.05.

Furthermore, an assessment of publication bias was conducted to discern any potential skew in the literature towards the publication of studies with significant findings. This involved the utilization of a funnel plot, allowing visual inspection of the distribution of effect sizes, with asymmetry potentially suggestive of publication bias. Additionally, the Egger regression test was employed to formally evaluate funnel plot asymmetry, determining whether the intercept of the regression line significantly deviated from zero, thus indicating the presence of a publication bias.

## Results

### Search Results

**Fig. 1:**
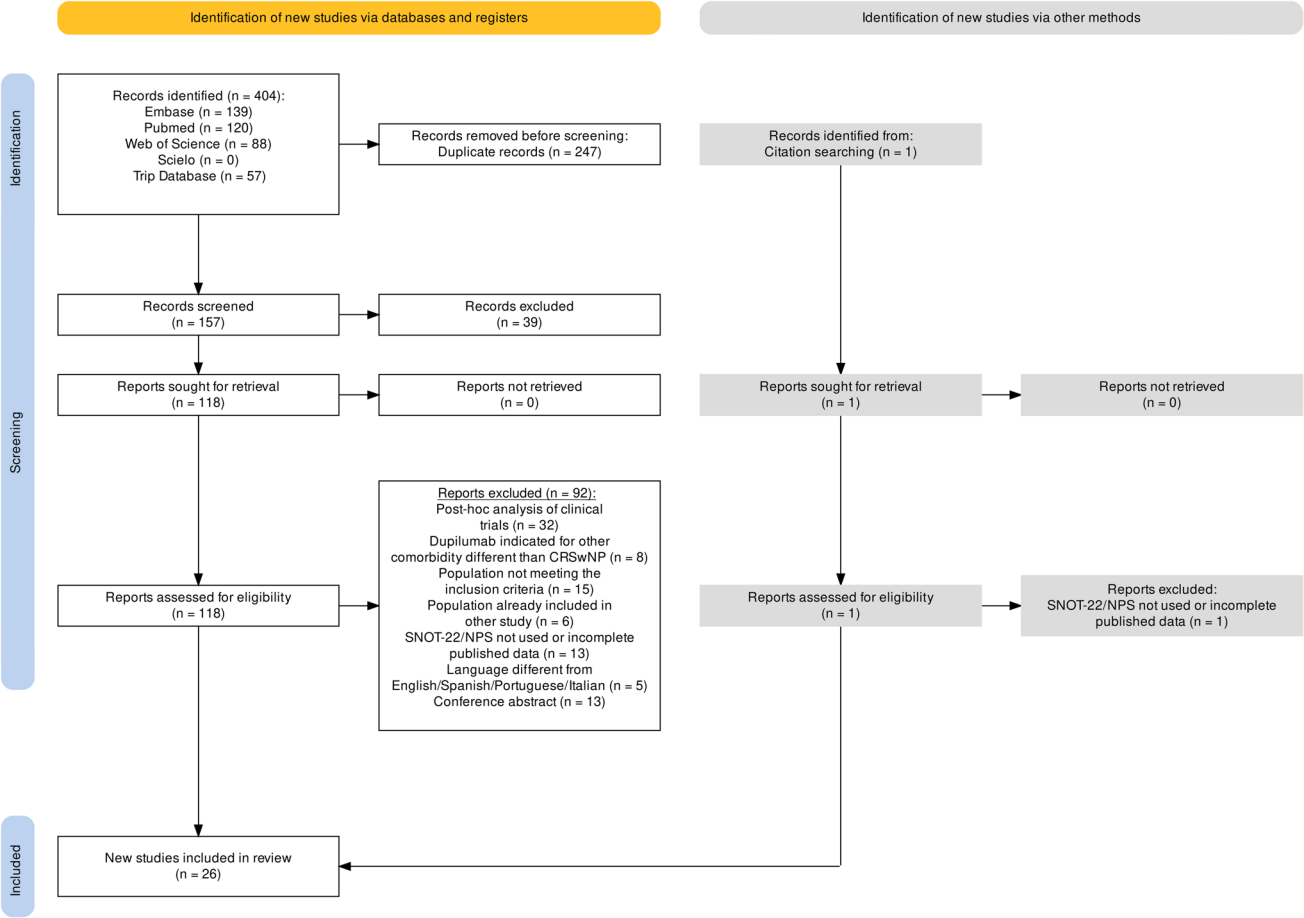
PRISMA flow chart of the search process

The PRISMA flow chart of the search process is shown in Fig. 1. The initial search returned 404 publications. After screening, 247 duplicated records were removed. Finally, after screening and complete reading, a total of 26 studies comprising 2,183 patients met the inclusion criteria.

Thirteen authors were contacted twice by email to request missing or unpublished data [72, 76, 77, 81, 84, 88, 89, 101, 103, 117–120]. Out of those thirteen, only three answered [79, 119, 120]. 

Of the selected articles, 92 publications were excluded following the exclusion criteria outlined above since they consisted of post hoc analysis of clinical trials (*n* = 32), conference abstracts (*n* = 13), studies where dupilumab was indicated for other comorbidity different from CRSwNP (*n* = 8), population not meeting the inclusion criteria (*n* = 15), population already included in other studies (*n* = 6), studies where SNOT-22 or NPS data were not used or were published incomplete (*n* = 13), and papers in different language other than English/Spanish/Portuguese/Italian (*n* = 5). References of excluded papers can be found in *Supplementary Annex* 1.

### Results of the Included Studies

The mean difference and standard deviation of the difference for SNOT-22 were estimated from medians and quantiles in 24 studies [24, 79, 86, 108, 117–136]. The same parameters were estimated for NPS in twenty-two [24, 108, 117–120, 122–126, 128, 129, 131, 132, 134–140]. 

#### General Results

Results are summarized in Table 1. The mean and SD for sample size is 83.8 ± 125.8 among the studies. The largest sample size from a RWE was reported by De Corso et al. [118] (648 patients) and the smallest by Piazzetta et al. [125] (14 patients).

**Table 1:** Description of the included studies (cont)

Author (Year)	Design / Level of evidence	Sample size and sex	Age. Mean ± SD (range)	Asthma (%)	N-ERD (%)	ESS (%)	Main outcome				Follow- up (weeks)
							Variable	T0 (mean ± SD)	T1 (mean ± SD)	Variation (mean ± SD)	
Cantone (2022)	Retrospective cohort study	53 (33 M; 20 F)	53.07 ± 12.74	70	21	100	**SNOT-22**	55.6 ± 15	15.9 ± 9.8	39.70 ± 2.41	25.8
De Corso (2023)	Retrospective cohort study	648 (400 M; 248 F)	54 ± 13.37 **	56.5	29.5	91.4	**SNOT-22**	58.75 ± 3.39	12.25 ± 2.42	46.50 ± 0.16	51.6
							**NPS **	5.75 ± 0.16	1 ± 0.32	4.75 ± 0.01	
							**Sniffin’s Sticks (0–16)**	4.25 ± 0.81	11.75 ± 0.81	7.50 ± 0.05	
							**Smell VAS **	8.2 ± 0.84	2.25 ± 0.81	5.95 ± 0.05	
Trimarchi (2022)	Prospective cohort study	21 (16 M; 5 F)	47 ± NR (29–84) 51.75 ± 14.75**	71.4	42.9	100	**SNOT-22**	62.33 ± 20.67	15.67 ± 18.29	46.66 ± 6.01	25.8
							**NPS**	5.33 ± 0.80	1.67 ± 2.39	3.66 ± 0.49	
							**B-SIT**	3.67 ± 1.59 **	8 ± 1.99 **	4.33 ± 0.55	
							**Smell VAS**	0	8 ± 1.59 **	- 8 ± 0.25	
Mocellin (2023)	Retrospective cohort study	23 (9 M; 14 F)	55.8 ± 14.8	82.6	65.2	73.9	**SNOT-22**	62.7 ± 18.6	29.6 ± 17.56	33.10 ± 5.33	25.8
							**NPS**	6.09 ± 1.31	2.73 ± 2.31	3.36 ± 0.53	
							**Sniffin’s Sticks** **(0–16)**	4 ± 2.72	9.6 ± 3.54	3.60 ± 0.71	
Jansen (2023)	Retrospective cohort study	40 (18 M; 22 F)	52.7 ± 15.3 (20 – 84)	88	52.5	100	**SNOT-22**	60.48 ± 22.17	20.8 ± 17.7	39.68 ± 4.46	55.9
							**NPS (0 – 8)**	4.30 ± 1.47	1.4 ± 1.1	2.90 ± 0.29	
							**Sniffin’s Sticks** **(0–12)**	3.22 ± 3.74	7.8 ± 3.5	4.58 ± 0.81	
Albrecht (2023)	Prospective cohort study	68 ( 36 M; 32 F)	49.81 ± 12.58	77.94	44.12	100	**SNOT-22**	53.74 ± 17.62	22.85 ± 16.66	30.89 ± 2.94	51.6
							**NPS (0 – 8)**	5.44 ±1.79	1.41 ± 1.54	4.03 ± 0.29	
							**Sniffin’s Sticks** **(0–12)**	2.26 ± 2.72	7.82 ± 3.51	5.56 ± 0.53	
							**Smell VAS**	9.16 ± 1.85	3.09 ± 2.73	6.07 ± 0.39	
Piazetta (2023)	Retrospective cohort study	14 (11 M; 3 F)	60.57 ± 12.31 (31 – 79)	57.1		92.85	**SNOT-22**	53.64 ± 22.39	11.86 ± 8.73	41.78 ± 5.88	24
							**NPS**	6.36 ± 1.28	2.63 ±1.33	3.73 ± 0.49	
La Mantia (2023)	Prospective controlled study	60 (38 M; 22 F)	50.83 ± 14.10	55	20	NR	**SNOT-22**	59.68 ± 21.11	16.21 ± 13.77	43.47 ± 3.18	25.8
							**NPS**	5.72 ± 1.24	2.2 ± 1.91	3.52 ± 0.29	
							**Sniffin’s Sticks** **(0–16)**	3.08 ± 2.63	10.23 ± 4.21	7.15 ± 0.62	
Grose (2023)	Retrospective cohort study	27 (15 M; 12 F)	43 ± 10.9	96.3	40.7	93	**SNOT-22**	60.6 ± 18.8	26.1 ± 17.9	34.50 ± 4.99	51.6
Orlando (2023)	Prospective controlled study	26 (20 M; 6 F)	53.9 ± NR (28–75)	53.8	26.2	92.3	**SNOT-22**	51.12 ± 19.50	23.19 ± 18.37	27.93 ± 5.25	51.6
							**NPS**	4.96 ± 2.13	3.65 ± 1.71	1.31 ± 0.53	
							**Sniffin’s Sticks** **(0–16)**	5.04 ± 2.94	9.59 ± 3.21	4.55 ± 0.85	
Kilty (2022)	Retrospective cohort study	53 ( 29; 24 F)	52.94 ± 10	88.67	37.73	90.56	**SNOT-22**	60.56 ± 21.63	23.47 ± 17.66	32.85 ± 21.10	28
Brkic (2023)	Retrospective cohort study	65 (42 M; 23 F)	51.3 ± 12.7	63.1	49.2	100	**NPS**	4.3 ± 1.9	1.2 ± 1.6	3.1 ± 1.7	25.8
Tsunemi (2023)	Retrospective cohort study	20 (13 M; 7 F)	50.6 ± 14.0	90	35	100	**NPS**	6.0 ± 0.2	0.2 ± 0.7	5.80 ± 0.14	78.69
Van der Lans (2023)	Prospective cohort study	228 (143 M; 85 F)	51 ± NR (18 – 90)	80.7	40.6	99.5	**SNOT-22 **	53.6 ± 19.6	21.2 ± 15.6	32.40 ± 1.65	96
							**NPS **	5.3 ± 1.9	1.3 ± 1.7	1.31 ± 0.53	
							**Sniffin’s Sticks** **(0–12)**	3.7 ± 2.4	7.3 ± 3.0	3.60 ± 0.25	
Böscke (2023)	Retrospective cohort study	41 (23 M; 18 F)	52.12 ± NR (27–79)	68.3	41.5	100	**SNOT-22**	51.59 ± 22.16	16.59 ± 11.92	35.00 ± 3.76	51.6
							**NPS**	4.88 ± 2.06	1.52 ± 1.75	3.36 ± 0.42	
							**Sniffin’s Sticks** **(0–12)**	2.86 ± 1.64	8.16 ± 2.75	5.30 ± 0.48	
Galletti (2023)	Prospective cohort study	170 (109 M; 61 F)	54 (45–63)	45.3	27.6	78.8	**SNOT-22**	65 ± 18.59	22.54 ± 13.85	42.46 ± 1.76	51.6
							**NPS **	5.96 ± 1.28	2.17 ± 1.81	3.79 ± 0.17	
							**Sniffin’s sticks** **(0 - 16)**	2.59 ± 2.74	10.99 ± 4.1	8.40 ± 0.37	
Alicandri-Ciufelli (2023)	Retrospective cohort study	145 (145 M; 89 F)	55.1± NR (27–86)	75.8	29.6	100	**SNOT-22**	56.1 ± 18.4	12.5 ± 9.4	43.60 ± 1.63	51.6
							**NPS **	5.6 ± 1.3	1.4 ± 1.6	4.20 ± 0.17	
							**Sniffin’s Sticks** **(Scale NR)**	5.7 ± 2.7	11.1 ± 2.4	5.40 ± 0.30	
							**Smell VAS **	7.4 ± 2.9	2.4 ± 2.7	5.00 ± 0.33	
Ferri (2023)	Prospective cohort study	29 (13 M; 16F)	54.0 ± 9.8	82.8	37.5	86.2	**SNOT-22**	62.5 ± 17.0	32.2 ± 18.0	30.30 ± 4.60	12.9
							**NPS**	6.4 ± 1.3	3.1 ± 2.0	3.30 ± 0.43	
Campion (2023)	Retrospective cohort study	97 (61 M; 36 F)	46.3 ± 14.2	64.9	51.5	92.8	**SNOT-22**	33.29 ± 23.04	13.34 ± 14.18	19.95 ± 2.67	25.8
							**NPS**	3.86 ± 2.25	1.28 ± 1.61	2.58 ± 0.28	
							**Sniffin’s sticks** **(0 - 12)**	5.92 ± 4.09	9.61 ± 3.10	3.69 ± 0.52	
Paoletti (2023)	Prospectively cohort study	33 (13 M; 20 F)	54.2 ± 11.2	87.9	60.6	78.8	**SNOT-22**	66.8 ± 15.1	38.4 ± 18.4	28.40 ± 4.12	4
							**NPS**	6.5 ± 1.4	4.3 ± 2.1	2.20 ± 0.43	
							**Smell VAS**	9.8 ± 0.8	5.6 ± 3.5	4.20 ± 0.53	
Giombi (2024)	Retrospective cohort study	53 (29 M; 24 F)	54.45 ± 9.87	30.2	11.3	90.6	**SNOT-22**	63.92 ± 19.98	27.96 ± 18.80	35.02 ± 21.03	12
							**NPS**	6.06 ± 1.51	3.04 ± 1.85	3.02 ± 0.33	
							**Smell VAS**	9.48 ± 1.74	4.70 ± 3.43	4.78 ± 0.50	
Garvey (2024)	Retrospective cohort study	39 (14M; 25 F)	52.21 ± 15.52	100	25.6	92.3	**SNOT-22**	57.34 ± 22.45	24.24 ± 22.45	33.10 ± 5.08	51.6
							**NPS**	4.15 ± 2.53	0.91 ± 1.99	3.24 ± 0.51	
Gal (2024)	Retrospective cohort study	47 (36 M; 11 F)	52.9 ± 13.5	74.5%	53.2	100	**SNOT-22**	52.4 ± 24.3	12.7 ± 10.5	39.7 ± 3.59	51.6
							**NPS**	6.15 ± 1.71	1.57 ± 1.40	4.58 ± 0.32	
							**Sniffin’s Sticks** **(0 - 16)**	1.6 ± 2.8	9.1 ± 5.4	7.50 ± 2.96	
Gelardi (2024)	Retrospective cohort study	27 (19 M; 8 F)	56.37 ± 10.09	63 %	NR	NR	**SNOT-22**	65 ± 22.12 **	12.83 ± 2.77 **	52.17 ± 3.67	51.6
							**NPS**	5.67 ± 1.58 **	2.17 ± 1.98 **	3.50 ± 0.52	
Sarnoch (2024)	Prospective cohort study	104 (51 M; 23 F)	50.3 ± 13.8	83 %	48	NR	**SNOT-22**	60.42 ± 19.36	28.71 ± 22.87	31.71 ± 2.93	94.6
							**NPS**	4.72 ± 1.60	0.86 ± 0.90	3.86 ± 0.17	
							**Sniffin’s Sticks** **(0 - 12)**	3.22 ± 3.65	9.67 ± 2.07	6.45 ± 0.40	
De Corso (2024)	Retrospective cohort study	52 (30 M; 22 F)	50.1 ± 13.6	69.2 %	26.9	86.5	**SNOT-22**	58.6 ± 18.8	12.2 ± 7.9	46.40 ± 2.62	51.6

*NA* (not applicable). *NR* (not reported). *SD* (standard deviation). *ESS* (endoscopic sinus surgery). *CRSwNP* (chronic rhinosinusitis with nasal polyps). *N-ERD* (NSAID – Non steroidal anti-inflammatory drugs – Exacerbated Respiratory Disease). *SNOT-22* (SinoNasal Outcome Test – 22. Score: 0 - 110). *NPS* (Nasal Polyp Score. Score: 0 - 8). *VAS* (Visual Analogue Scale. Scor: 0 – 10 cm). *B-SIT* (Brief Smell Identification Test. Score: 0 – 12). ** Mean and Standard Deviation estimated using Wan’s method.

The weighted mean and SD for age was 52.6 ± 3.3 years. The lowest was reported by Grose et al. [127] (43 years) and the highest one by Piazzetta et al. [125] (60.6 years).

The weighted mean and SD for follow up time was 51.9 ± 23.2 weeks, being the lowest 4 weeks (Paoletti et al. [131]) and the highest 96 (Van der Lans et al. [128]).

The weighted mean and SD for the proportion of patients suffering from asthma was 66.7 ± 14.5%, being the lowest 30.2% (Giombi et al. [132]) and the highest 100% (Garvey et al. [138]).

The weighted mean and SD for the proportion of patients suffering from N-ERD was 44.6 ± 14.5%, being the lowest 11.3% (Giombi et al. [132]) and the highest 65.2% (Mocellin et al. [122]).

#### SNOT-22

The results are summarized in Table 1. Thirty-one observational RWE assessed SNOT-22 [24, 77, 81, 84, 88, 89, 92, 93, 108, 117–136, 138, 139]. 

Twenty-four of them could be included in the meta-analysis. Bellochi et al. [81], Ottaviano et al. (2022) [77], Ottaviano et al. (2023) [89] and Brkic et al. [137] could not be included because they did not provide the standard deviation (of the difference or before and after treatment). Haxel et al. [88] could not be included because they only provided the mean value at baseline. Nettis et al. [84], Ottaviano et al. (2024) [93] and Riva et al. [92] could not be included, as they provided data measured in the median and interquartile range without the necessary data (minimum and maximum or first and third quartile) to estimate the mean and standard deviation following Wan’s method [116]. 

**Fig. 2:**
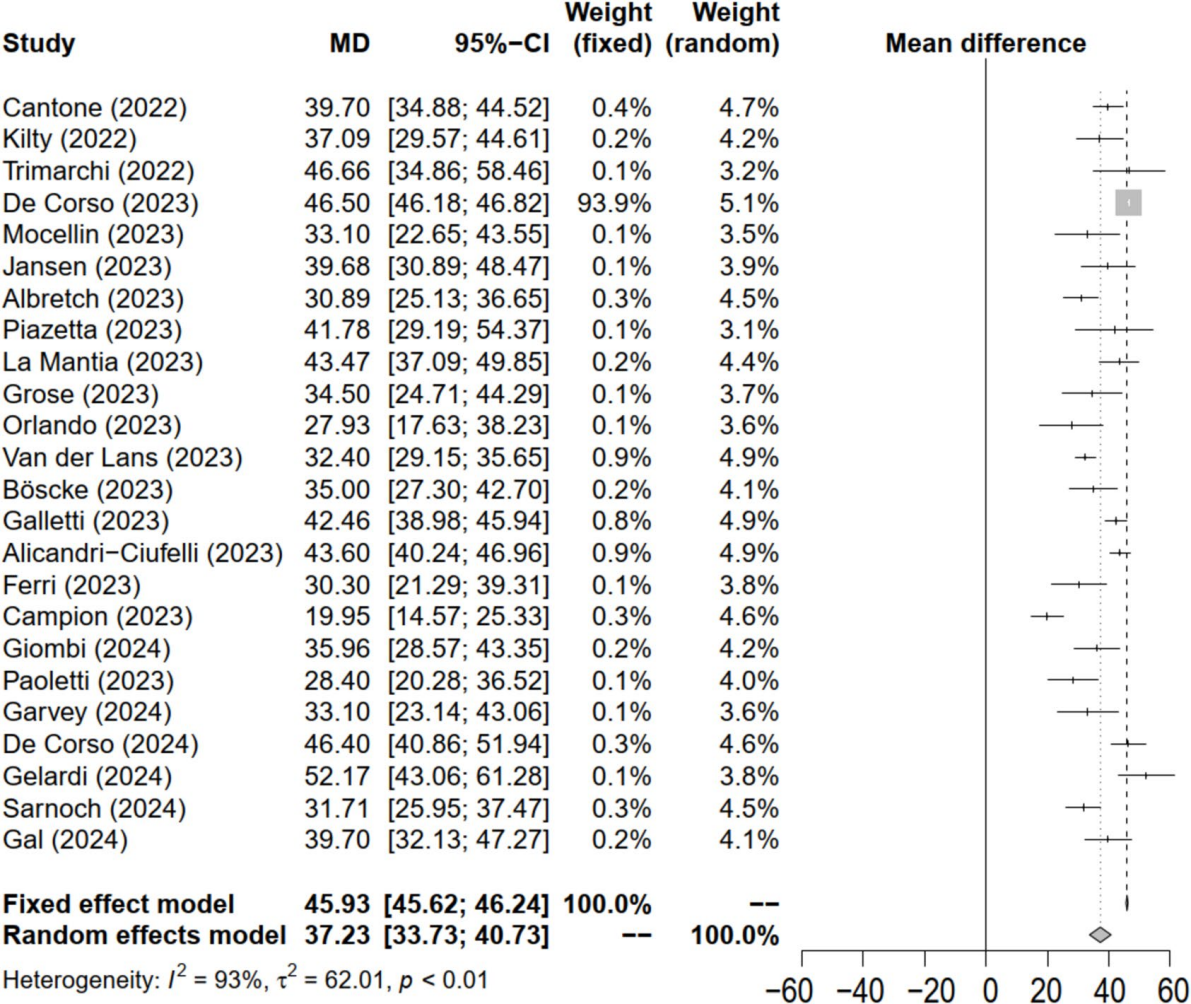
Forest plot for the SNOT-22 difference after dupilumab treatment. Statistical significance was found, since the diamond did not reach the vertical discontinuous line. Taking into account the high heterogeneity observed, the random effects model was assumed

Twenty of them originally provided their data as mean and standard deviation [24, 108, 119–133, 135, 136, 138], one author provided them under request [139] and Wan’s method [116] could be applied for estimating data for three studies [117, 118, 134]. This way, changes in SNOT-22 for 2094 patients could be combined in a meta-analysis (Fig. 2).

Since the I^2^ heterogeneity coefficient was 96%, a random-effects model was assumed, being the mean SNOT-22 difference after dupilumab treatment 37.2 (Fig. 2).

**Fig. 3:**
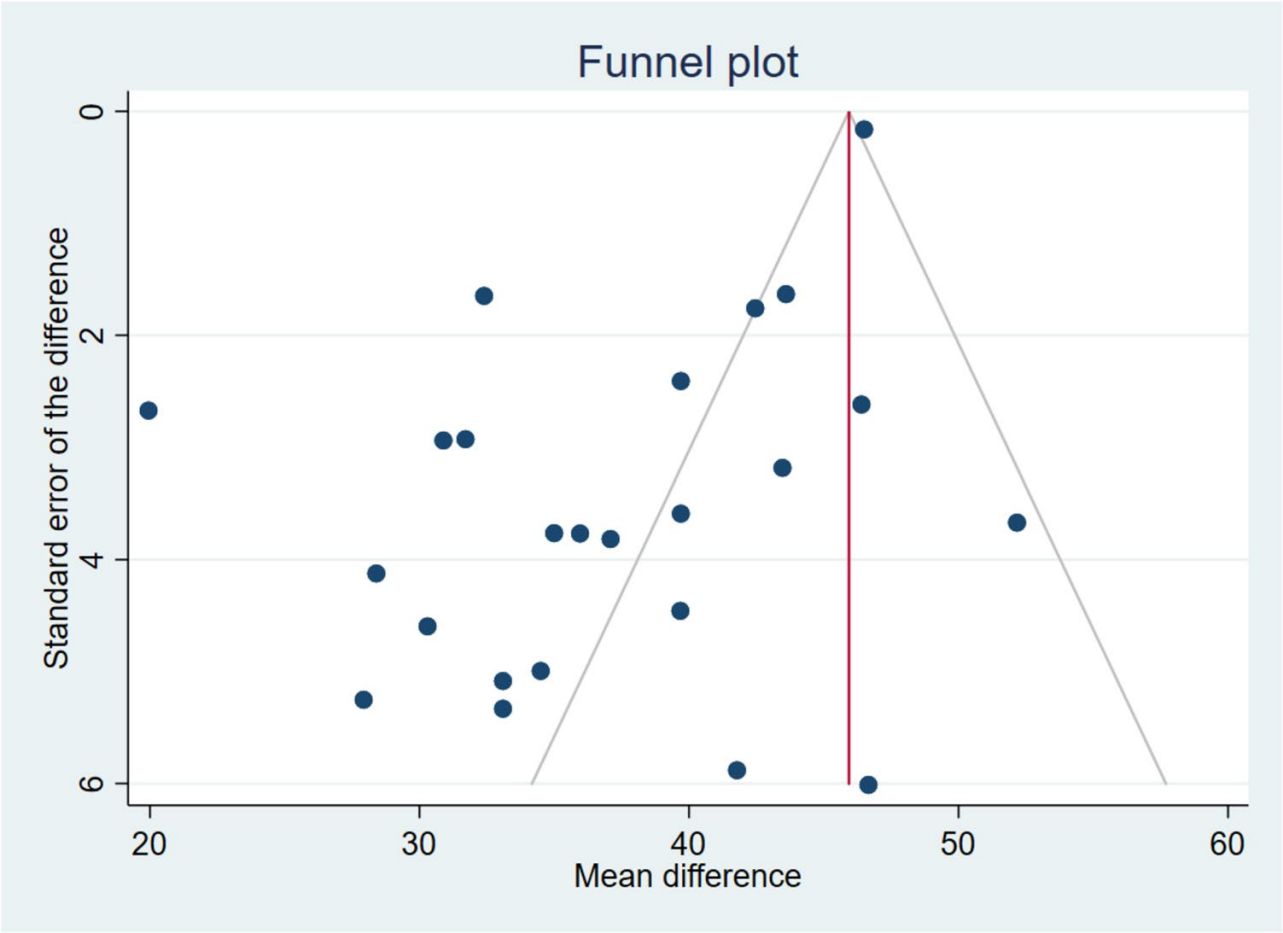
Funnel plot of the SNOT-22 difference after dupilumab treatment. Most of the published studies are located on the left part of the graph bias favoring the studies with results inferior to the mean

An Egger test was performed to investigate the possibility of publication bias, yielding a coefficient of −1.03 (*p* < 0.30). Figure 3 displays the Funnel plot for the difference in SNOT-22 scores among the published studies.

When comparing the results of the current study with the results provided by the SINUS-52 [141] (28.5 ± 2.2) clinical trial using a student T test for independent samples, statistically significant differences were found with the fixed effect model (t = 95.3; *p* < 0.01) as well as with random-effect model (t = 46.7; *p* < 0.01) favoring RWE.

#### Nasal Polyp Score (NPS)

The results are summarized in Table 1. Thirty observational RWE assessed NPS [24, 108, 117–120, 122–126, 128, 129, 131, 137, 139, 140]. 

Eight of them [77, 81, 84, 88, 89, 92, 93, 133] could not be included in the meta-analysis because of the same reasons stated above regarding SNOT-22 values.

**Fig. 4:**
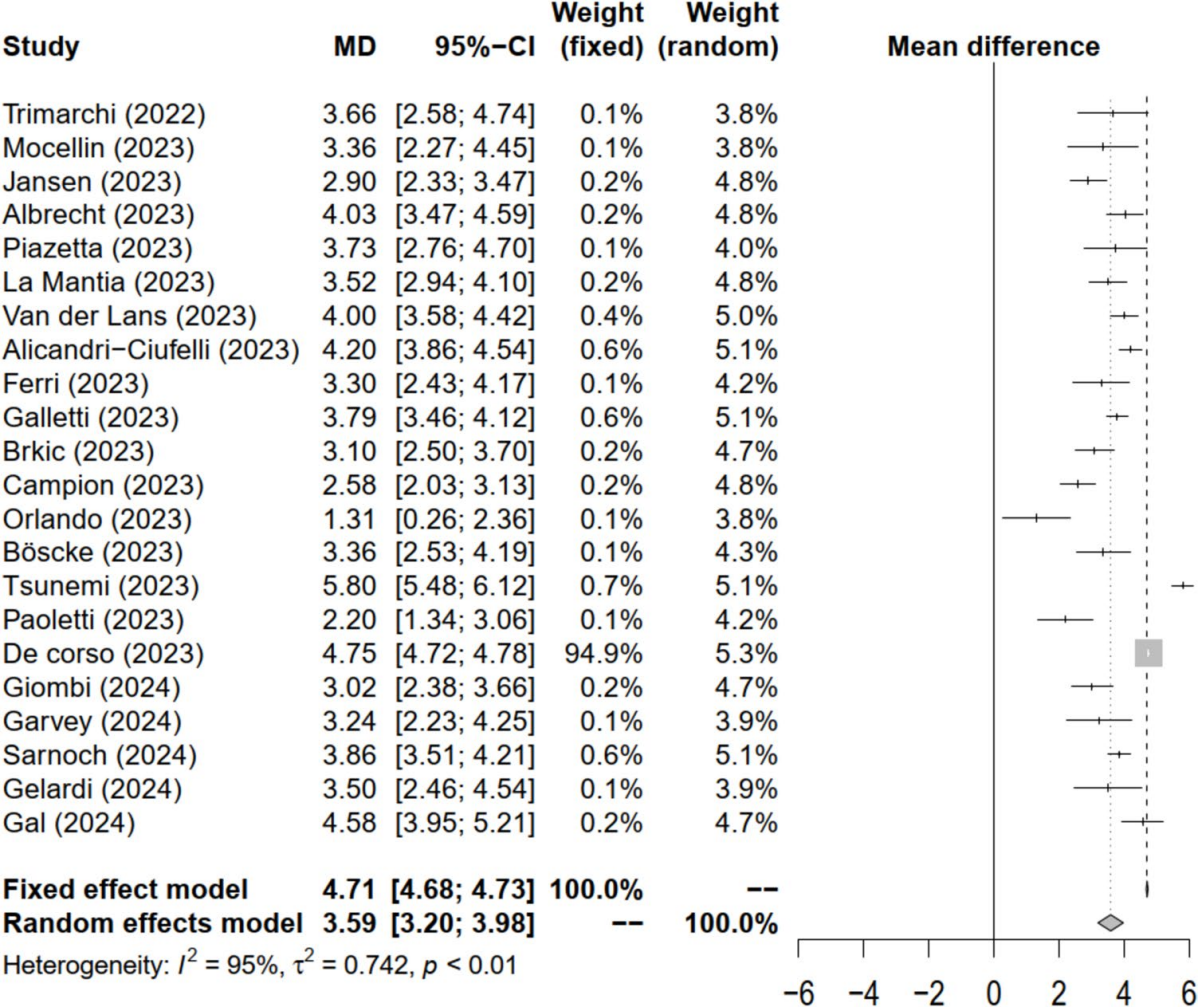
Forest plot for the difference in Nasal Polyp Size (NPS) after dupilumab treatment. Statistical significance was found, since the diamond did not reach the vertical discontinuous line. Taking into account the high heterogeneity observed, the random effects model was assumed

Fifteen of them originally provided their data as mean and standard deviation [24, 108, 119–131], one author provided them on request [139] and Wan’s method [116] could be applied for estimating data for three studies [117, 118, 134]. In this manner, changes in NPS for 1994 patients could be combined in a meta-analysis (Fig. 4).

The heterogeneity coefficient was 96%, suggesting the high heterogeneity of samples. The mean difference in NPS after dupilumab treatment under a random effects model was 3.6 (Fig. 4).

Comparing the results of the current study with those resulting from the SINUS-52 clinical trial [141] using a student t test for independent samples, statistically significant differences were found with the fixed-effects model (t = 146.9; *p* < 0.01) as well as with the random-effects model (t = 75.6; *p* < 0.01).

**Fig. 5:**
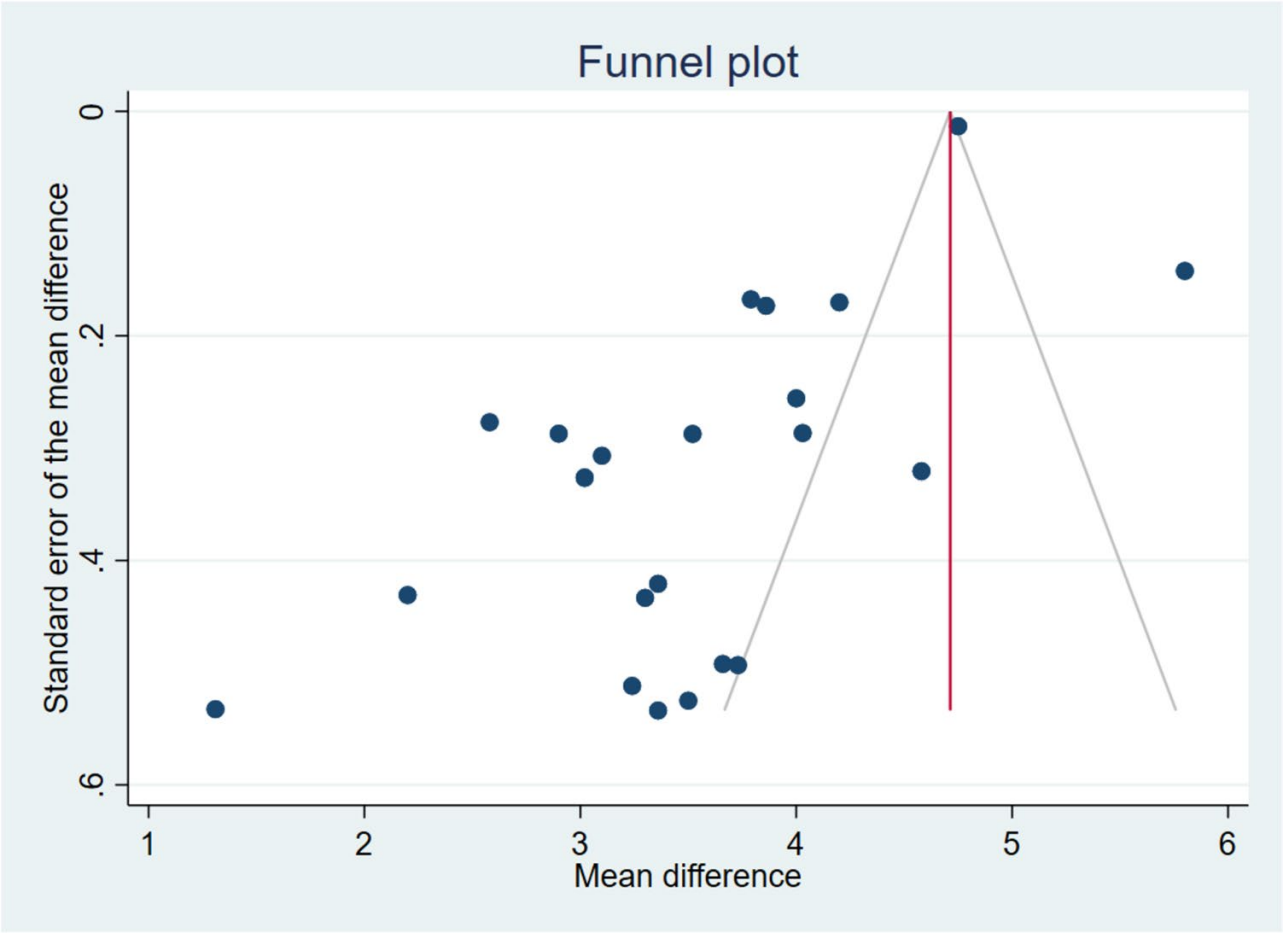
Funnel plot of the Nasal Polyp Size (NPS) difference after dupilumab treatment. Most of the published studies are located on the left part of the graph bias favoring the studies with results inferior to the mean

An Egger test was performed to investigate the possibility of publication bias, yielding a coefficient of −3.35 (*p* < 0.001). Figure 5 displays the Funnel plot for the difference in NPS scores among the published studies.

## Discussion

To the best of our knowledge, this is the first meta-analysis evaluating nasal variables improvement after dupilumab treatment in RWE performed on patients where the indication for treatment was severe CRSwNP. This review concludes that dupilumab is a promising therapy for patients with CRSwNP, with results in real life that outweigh those reported in clinical trials.

Several published studies have measured the nasal effects of dupilumab in patients with CRSwNP. However, only in a small fraction of these studies, severe CRSwNP has been the indication for the biologic therapy, as in the vast majority it has been indicated for other conditions such as severe asthma, or eosinophilic esophagitis, among others. As mentioned above, these studies were excluded in our review (*Supplementary Annex* 1).

The goals of CRSwNP treatment are to achieve effective and sustained symptom control, minimize polyp recurrence, and control of comorbid lower airway disease while minimizing the risk of side-effects associated with systemic corticosteroid use and revision ESS [20]. In our research, we decided to focus on quantifying the variability in nasal symptoms using the SNOT-22 questionnaire as our primary target. A total of 24 studies could finally be combined in a meta-analysis in which the main result to highlight is that RWE have shown satisfactory results in nasal symptoms, better than those reported in the SINUS-52 RCT [141]. SINUS-52 obtained a decrease of 28.5 points on the SNOT-22 in the treatment cohort, whereas our meta-analysis obtained 37.2 points of decrease under a random effects model. It represents an improvement of 4.18 times the minimal clinically importance difference (MCID), which was established at 8.9 points for SNOT-22 [49]. In SINUS-52 it was 3.2 times the MCID [141]. 

Our second endpoint was the NPS. The meta-analysis suggests better outcomes in terms of NPS decrease for RWE compared to the SINUS-52 clinical trial [141]. SINUS-52 obtained a decrease of 2.3 ± 0.2 points in the NPS in the treatment cohort, while the present meta-analysis obtained 3.6 ± 0.2 points of decrease. These results could be interpreted as the SINUS-52 clinical trial does not have adequate external validity, with better results in real-life practice. External validity is a problem for several RCT as they try to achieve an accurate and homogeneous patient selection (internal validity), which does not usually correlate with real practice (external validity) [142]. 

It is important to highlight the analysis regarding the bias of publication performed in this study. In relation to SNOT-22, the graphic representation (with funnel plot) could suggest that a publication bias exists. Nevertheless, when performing the Egger regression to estimate it, a *p* > 0.3 is obtained, indicating that there is not a publication bias. On the other hand, a publication bias exists in relation to NPS. However, it is also of interest to consider that this is a negative publication bias. As observed in the funnel plot, it is likely that there are yet-to-be-published articles in which a greater reduction in NPS could be found, indicating that the difference posttreatment is probably underestimated. In this case, RWE is better than the reported in the SINUS-52 clinical trial [141]. However, being scientifically prudent, both cohorts should not be fully compared, as they encompass different types of patients. In the SINUS-52 RCT cohort, the mean was 50.2 points for baseline SNOT-22 and 6.1 for baseline NPS, while the weighted mean in our meta-analysis was 57.3 points for baseline SNOT-22 and 5.5 for baseline NPS.

In relation to the comorbidities, proportions are different as well. In the SINUS-52 RCT [141], 57% and 23% of the patients suffered from asthma and N-ERD, respectively. The weighted mean for the proportion of asthmatic patients in the studies included in our meta-analysis was 66.7% for asthma and 44.6% for N-ERD. This relationship may be highly relevant when evaluating the response to treatment. Patients diagnosed with CRSwNP and concurrent asthma, with or without N-ERD, experience a more severe form of the disease. This is characterized by an elevated nasal polyp growth, higher rate of recurrence after surgery, frequent reliance on systemic corticosteroids, inadequate asthma control, and increased healthcare costs and resource utilization [143]. Even so, it should be mentioned that asthma control appears to be improved, as some studies found a statistically significant improvement in the Asthma Control Test after 1 and 3 months post-treatment [108, 131]. Following this point, an additional potential confounding factor is the prevalence of severe asthma; although most authors provide the prevalence of asthma, they do not specify the severity of the disease or its control degree.

The loss of smell, which is one of the most challenging symptoms for patients with severe CRSwNP, is associated with both the severity and recurrence of the disease significantly affecting their QoL [127, 128]. Its recovery is one of the first signs of treatment efficacy that patients experience once dupilumab therapy is started [24]. There is a lack of information on whether a history of previous surgery influences the speed of recovery. Among the studies included in the revision, seventeen of them measured smell function [24, 79, 117–120, 122–124, 126, 128, 129, 132, 133, 135, 136]. Furthermore, the wide variety of methods used to measure olfactory impairment (Sniffin’ Sticks – 16 [79, 118, 120, 122, 126, 133, 136], Sniffin’ Sticks–12 [24, 119, 123, 124, 128, 135], Visual Analog Scale [117, 118, 124, 129, 131–133], Brief Smell Identification Test [117]) makes it difficult to obtain comparable data. A post hoc analysis of SINUS-24 and SINUS-52 cohorts, found that patients with three or more previous ESS at baseline, exhibited the worst results regarding olfaction [49]. However, they showed similar improvement during the follow-up period regardless of the number of prior ESS without reporting any significant correlation between the results and the number of previous ESS. In this regard, De Corso et al. claimed that olfaction improved faster in patients without previous surgeries, but that difference was not clinically relevant [118].

Another factor to be considered is the history of previous surgery, as it could interfere with the speed of improvement. In the ESS sinus surgery at the beginning of the study. The weighted mean with previous surgeries among the patients included in our meta-analysis is 77.8%, being the range from 56.5 to 100% in eight studies [24, 117, 121, 123, 124, 129, 137, 140]. In this sense, some studies phrased that SNOT-22 and NPS showed a faster decrease in patients who had undergone previous surgery [118, 122]. Although most of the reviewed studies did not analyze this factor in RWE, a post hoc analysis described how a short onset of biological treatment (< 3 years since the last ESS) correlated with greater improvements in endoscopic findings [49], highlighting the importance of timing in combined treatment. In addition to the surgical history, it would also be necessary to include the type of surgery performed, since extensive surgeries appear to achieve better results regarding illness control [118, 122, 144]. The majority of authors utilized different systems to classify the surgeries performed, making comparisons of the surgery-related outcomes challenging. Nevertheless, a more comprehensive surgical analysis is necessary in this fasting moving field.

Although the results found in this study seem to be better than those obtained in RCT, there are several limitations that should be considered. The first limitation is the novelty of this treatment. In medicine, first reports tend to be the most notable, whereas subsequent studies, as enthusiasm decreases, tend to diminish. As all included studies were published between 2022 and 2023, we cannot know if we are seeing these first results. At present, the cumulative meta-analysis does not suggest a latency in published results, but future research including new studies is highly recommended to study this phenomenon. Another limitation lies in the variability of the follow-up times. Although it may not be the best option to merge studies with such marked variability in follow-up time in a meta-analysis, it should be taken into account that the most significant reduction was observed in the initial weeks, with a slow and gradual improvement afterwards, particularly for subjective parameters such as SNOT-22, VAS scores for loss of smell and nasal obstruction. For this reason, and given the available evidence at this moment, it seems reasonable to combine the results. A stratified analysis by subgroups should be performed in future studies as more samples become available.

Despite this systematic review with meta-analysis follows rigorous guidelines to evaluate the efficacy of dupilumab therapy, there are limitations. Strict inclusion criteria, which exclude small sample sizes, certain specific study designs and those studies where dupilumab was indicated for other conditions (*Supplementary Annex* 1), may have led to omitted data. In addition, methodological difficulties in estimating the mean and standard deviation from partially published or median-based data, as well as the conversion of follow-up periods into weeks, introduce uncertainties. Thus, although these inherent limitations of the review are attempted to be controlled by a meticulous statistical analysis process, controlling for heterogeneity and publication biases of the included articles, cautious interpretation and further research are warranted to validate the findings.

Despite the limitations discussed above, it should be kept in mind that we are currently dealing with a new line of treatment. Therefore, it is to be expected that the available evidence presents these types of limitations. This kind of systematic review, which gathers all the available evidence, helps to shed some light and limit the potential distortion of the results generated by small observational studies. It also highlights pitfalls and knowledge gaps to guide future studies.

## Conclusion

The available evidence is limited by the observational design of the included studies, and any results should be carefully managed. The available evidence appears to favor dupilumab RWE studies compared with the previous dupilumab RCT (SINUS-52), with a better response regarding NPS and SNOT-22.

## Key References

- De Corso E, Pasquini E, Trimarchi M, et al. (2023) Dupilumab in the treatment of severe uncontrolled chronic rhinosinusitis with nasal polyps (CRSwNP): A multicentric observational Phase IV real-life study (DUPIREAL). Allergy 78:2669–2683. 10.1111/all.15772.
  - COMMENT: This RWE study has the biggest sample size among the included into the meta-analysis.
- Alobid I, Colás C, Castillo J, et al. (2023) Spanish Consensus on the Management of Chronic Rhinosinusitis With Nasal Polyps (POLIposis NAsal/POLINA 2.0). J Investig Allergol Clin Immunol 33:317–331. 10.18176/jiaci.0910.
  - COMMENT: The POLINA consensus provides new definitions of control, therapeutic management (including surgery and evaluation of severity), indications for use of biologics, and response.
- Fokkens WJ, Viskens A-S, Backer V, et al. (2023) EPOS/EUFOREA update on indication and evaluation of Biologics in Chronic Rhinosinusitis with Nasal Polyps 2023. Rhinology 61:194–202. 10.4193/Rhin22.489.
  - COMMENT: Criteria for the selection of patients who would benefit from biologics were updated.

## Supplementary Information

Below is the link to the electronic supplementary material.

ESM 1 (DOCX 32.6 KB)

## Data Availability

No datasets were generated or analysed during the current study.
