# Supplementary material for: Effect of Dupilumab in CRSwNP Sinonasal Outcomes from Real Life Studies: A Systematic Review with Meta-analysis
Source: Curr Allergy Asthma Rep. 2025 Feb 5;25(1):13. doi: 10.1007/s11882-025-01192-y (PMC11799128; doi:10.1007/s11882-025-01192-y)
Supplement: Supplementary file 1 — (DOCX 32.6 KB) [file 11882_2025_1192_MOESM1_ESM.docx]

*Supplementary Annex 1 – Excluded studies*

*POST-HOC ANALYSIS OF CLINICAL TRIALS*

1. Laidlaw TM, Bachert C, Amin N, et al (2021) Dupilumab improves upper and lower airway disease control in chronic rhinosinusitis with nasal polyps and asthma. Ann Allergy Asthma Immunol 126:584-592.e1. https://doi.org/10.1016/j.anai.2021.01.012
2. Boguniewicz M, Beck LA, Sher L, et al (2021) Dupilumab Improves Asthma and Sinonasal Outcomes in Adults with Moderate to Severe Atopic Dermatitis. J Allergy Clin Immunol Pract 9:1212-1223.e6. https://doi.org/10.1016/j.jaip.2020.12.059
3. Miglani A., Soler Z.M., Smith T.L., et al (2023) A comparative analysis of endoscopic sinus surgery versus biologics for treatment of chronic rhinosinusitis with nasal polyposis. Int Forum Allergy Rhinol 13:116–128. https://doi.org/10.1002/alr.23059
4. Khan A.H., Reaney M., Guillemin I., et al (2022) Development of Sinonasal Outcome Test (SNOT-22) Domains in Chronic Rhinosinusitis With Nasal Polyps. Laryngoscope 132:933–941. https://doi.org/10.1002/lary.29766
5. Busse WW, Wellman A, Diamant Z, et al (2022) Impact of dupilumab on SNOT-22 sleep and function scores in CRSwNP. J Allergy Clin Immunol Pract 10:2479-2482.e3. https://doi.org/10.1016/j.jaip.2022.05.013
6. Weinstein SF, Katial R, Jayawardena S, et al (2018) Efficacy and safety of dupilumab in perennial allergic rhinitis and comorbid asthma. J Allergy Clin Immunol 142:171-177.e1. https://doi.org/10.1016/j.jaci.2017.11.051
7. Mullol J, Laidlaw TM, Bachert C, et al (2022) Efficacy and safety of dupilumab in patients with uncontrolled severe chronic rhinosinusitis with nasal polyps and a clinical diagnosis of NSAID-ERD: Results from two randomized placebo-controlled phase 3 trials. Allergy 77:1231–1244. https://doi.org/10.1111/all.15067
8. Bachert C., Zinreich S.J., Hellings P.W., et al (2020) Dupilumab reduces opacification across all sinuses and related symptoms in patients with CRSwNP. Rhinology 58:10–17. https://doi.org/10.4193/Rhin18.282
9. Busse WW, Pavord ID, Siddiqui S, et al (2023) Dupilumab Improves Outcomes in Patients with Chronic Rhinosinusitis with Nasal Polyps and Coexisting Asthma Irrespective of Baseline Asthma Characteristics. J Asthma Allergy 16:411–419. https://doi.org/10.2147/JAA.S391896
10. Desrosiers M, Mannent LP, Amin N, et al (2021) Dupilumab reduces systemic corticosteroid use and sinonasal surgery rate in CRSwNP. Rhinology 59:301–311. https://doi.org/10.4193/Rhin20.415
11. Bachert C, Khan AH, Hopkins C, et al (2022) Rapid and Continuing Improvements in Nasal Symptoms with Dupilumab in Patients with Severe CRSwNP. J Asthma Allergy 15:557–563. https://doi.org/10.2147/JAA.S355391
12. Fujieda S, Matsune S, Takeno S, et al (2021) The Effect of Dupilumab on Intractable Chronic Rhinosinusitis with Nasal Polyps in Japan. Laryngoscope 131:E1770–E1777. https://doi.org/10.1002/lary.29230
13. Lee SE, Amin N, Mannent LP, et al (2023) The relationship of sinus opacification, olfaction and dupilumab efficacy in patients with CRSwNP. Rhinology 61:531–540. https://doi.org/10.4193/Rhin22.220
14. Hopkins C, Wagenmann M, Bachert C, et al (2021) Efficacy of dupilumab in patients with a history of prior sinus surgery for chronic rhinosinusitis with nasal polyps. Int Forum Allergy Rhinol 11:1087–1101. https://doi.org/10.1002/alr.22780
15. Mullol J, Bachert C, Amin N, et al (2022) Olfactory Outcomes With Dupilumab in Chronic Rhinosinusitis With Nasal Polyps. J Allergy Clin Immunol Pract 10:1086-1095.e5. https://doi.org/10.1016/j.jaip.2021.09.037
16. Canonica GW, Bourdin A, Peters AT, et al (2022) Dupilumab Demonstrates Rapid Onset of Response Across Three Type 2 Inflammatory Diseases. J Allergy Clin Immunol Pract 10:1515–1526. https://doi.org/10.1016/j.jaip.2022.02.026
17. Chuang C.-C., Guillemin I., Bachert C., et al (2022) Dupilumab in CRSwNP: Responder Analysis Using Clinically Meaningful Efficacy Outcome Thresholds. Laryngoscope 132:259–264. https://doi.org/10.1002/lary.29911
18. Peters AT, Wagenmann M, Bernstein JA, et al (2023) Dupilumab efficacy in patients with chronic rhinosinusitis with nasal polyps with and without allergic rhinitis. Allergy Asthma Proc 44:265–274. https://doi.org/10.2500/aap.2023.44.230015
19. Gevaert P, Lee SE, Settipane RA, et al (2023) Dupilumab provides early and durable improvement of symptoms in patients with chronic rhinosinusitis with nasal polyps. Clin Transl Immunology 12:e1433. https://doi.org/10.1002/cti2.1433
20. Bachert C., Khan A.H., Lee S.E., et al (2023) Prevalence of type 2 inflammatory signatures and efficacy of dupilumab in patients with chronic rhinosinusitis with nasal polyps from two phase 3 clinical trials: SINUS-24 and SINUS-52. Int Forum Allergy Rhinol. https://doi.org/10.1002/alr.23249
21. Hellings P.W., Peters A., Chaker A.M., et al (2021) Rapid and sustained effects of dupilumab in patients with severe chronic rhinosinusitis with nasal polyps: Analysis of the sinus-24 and sinus-52 phase 3 trails. Am J Respir Crit Care Med 203:. https://doi.org/10.1164/ajrccm-conference.2021.203.1_MeetingAbstracts.A1345
22. Bachert C., Khan A., Fokkens W., et al (2022) Onset, Maintenance, and Durability of Response with Dupilumab in Chronic Rhinosinusitis with Nasal Polyps. Eur Respir J 60:. https://doi.org/10.1183/13993003.congress-2022.1711
23. Bachert C., Khan A., Lee S., et al (2022) Dupilumab improves chronic rhinosinusitis with nasal polyps disease outcomes irrespective of type 2 signature definition. Ann Allergy Asthma Immunol 129:S72–S73. https://doi.org/10.1016/j.anai.2022.08.710
24. Bachert C., Peters A.T., Heffler E., et al (2021) A responder analysis to demonstrate dupilumab treatment effect across objective and patient-reported endpoints for patients with severe chronic rhinosinusitis with nasal polyps (CRSwNP). Clin Exp Allergy 51:1665–1666. https://doi.org/10.1111/cea.14044
25. Busse W., Pavord I., Siddiqui S., et al (2021) Dupilumab improves crswnp/asthma outcomes in patients with crswnp and comorbid asthma irrespective of asthma characteristics. Ann Allergy Asthma Immunol 127:S52–S53. https://doi.org/10.1016/j.anai.2021.08.158
26. Laidlaw T.M., Mullol J., Fan C., et al (2019) Dupilumab improves nasal polyp burden and asthma control in patients with CRSwNP and AERD. J Allergy Clin Immunol Pract 7:2462-2465.e1. https://doi.org/10.1016/j.jaip.2019.03.044
27. Lane A., Mullol J., Hopkins C., et al (2022) Dupilumab leads to reduction of anosmia in patients with severe chronic rhinosinusitis with nasal polyps. J Allergy Clin Immunol 149:AB144. https://doi.org/10.1016/j.jaci.2021.12.486
28. Peters A., Wagenmann M., Bernstein J.A., et al (2021) Efficacy of dupilumab in patients with chronic rhinosinusitis with nasal polyps and allergic rhinitis. Am J Respir Crit Care Med 203:. https://doi.org/10.1164/ajrccm-conference.2021.203.1_MeetingAbstracts.A1340
29. Soler Z., Lane A., Patel Z., et al (2022) Association between smell loss, disease burden, and dupilumab efficacy in chronic rhinosinusitis with nasal polyps. Ann Allergy Asthma Immunol 129:S72. https://doi.org/10.1016/j.anai.2022.08.709
30. Hellings P., Bachert C., Mullol J., et al (2018) Dupilumab improves ACQ-5 items in CRSwNP patients with comorbid asthma. Respirology 23:153. https://doi.org/10.1111/resp.13268
31. Hopkins C, Mullol J, Khan AH, et al (2024) Impact of Dupilumab on Sinonasal Symptoms and Outcomes in Severe Chronic Rhinosinusitis With Nasal Polyps. Otolaryngol Head Neck Surg 170:1173–1182. https://doi.org/10.1002/ohn.627
32. Bachert C, Khan AH, Lee SE, et al (2023) Prevalence of type 2 inflammatory signatures and efficacy of dupilumab in patients with chronic rhinosinusitis with nasal polyps from two phase 3 clinical trials: SINUS-24 and SINUS-52. Int Forum Allergy Rhinol. https://doi.org/10.1002/alr.23249

*DUPILUMAB INDICATED FOR OTHER DISEASES DIFFERENT THAN CRSwNP*

1. Mustafa SS, Vadamalai K, Scott B, Ramsey A (2021) Dupilumab as Add-on Therapy for Chronic Rhinosinusitis With Nasal Polyposis in Aspirin Exacerbated Respiratory Disease. Am J Rhinol Allergy 35:399–407. https://doi.org/10.1177/1945892420961969
2. Napolitano M, Maffei M, Patruno C, et al (2021) Dupilumab effectiveness for the treatment of patients with concomitant atopic dermatitis and chronic rhinosinusitis with nasal polyposis. Dermatol Ther 34:e15120. https://doi.org/10.1111/dth.15120
3. Lyly A., Genberg E., Kauppi P., et al (2023) Real-Life Experience of Biologic Treatment for Asthma on Chronic Rhinosinusitis: A Finnish Cohort. Int Arch Allergy Immunol 184:149–160. https://doi.org/10.1159/000526365
4. Nolasco S., Campisi R., Cipolla A., et al (2022) Dupilumab effectiveness in patients with type 2-high severe asthma and chronic rhinosinusitis with nasal polyps. Eur Respir J 60:. https://doi.org/10.1183/13993003.congress-2022.3319
5. Förster-Ruhrmann U, Stergioudi D, Szczepek AJ, et al (2023) A real-life comparison of pulmonary and nasal outcomes in patients with severe asthma and nasal polyposis treated with T2-biologics. World Allergy Organization Journal 16:100746. https://doi.org/10.1016/j.waojou.2023.100746
6. Pelaia C, Lombardo N, Busceti MT, et al (2021) Short-Term Evaluation of Dupilumab Effects in Patients with Severe Asthma and Nasal Polyposis. J Asthma Allergy 14:1165–1172. https://doi.org/10.2147/JAA.S328988
7. Berger P, Menzies-Gow A, Peters AT, et al (2023) Long-term efficacy of dupilumab in asthma with or without chronic rhinosinusitis and nasal polyps. Ann Allergy Asthma Immunol 130:215–224. https://doi.org/10.1016/j.anai.2022.11.006
8. Caminati M, Maule M, Benoni R, et al (2024) Dupilumab Efficacy on Asthma Functional, Inflammatory, and Patient-Reported Outcomes across Different Disease Phenotypes and Severity: A Real-Life Perspective. Biomedicines 12:390. https://doi.org/10.3390/biomedicines12020390

*PATIENTS NOT MEETING THE INCLUSION CRITERIA*

1. Pelaia C, Benfante A, Busceti MT, et al (2023) Real-life effects of dupilumab in patients with severe type 2 asthma, according to atopic trait and presence of chronic rhinosinusitis with nasal polyps. Front Immunol 14:1121237. https://doi.org/10.3389/fimmu.2023.1121237
2. Gerstacker K., Ketterer M.C., Jakob T.F., Hildenbrand T. (2023) Real Life Observational Study of Treatment Success of Monoclonal Antibodies for Refractory Chronic Rhinosinusitis with Nasal Polyps. J Clin Med 12:4374. https://doi.org/10.3390/jcm12134374
3. Brkic FF, Liu DT, Klimbacher R, et al (2023) Efficacy and safety of switching between biologics in chronic rhinosinusitis with nasal polyps or N-ERD. Rhinology 61:320–327. https://doi.org/10.4193/Rhin22.408
4. Book R, Eligal S, Tal Y, Eliashar R (2023) Biological Treatment for Uncontrolled Chronic Rhinosinusitis with Nasal Polyps: Preliminary Real-World Results from a Tertiary Medical Center. J Clin Med 12:. https://doi.org/10.3390/jcm12113671
5. De Corso E, Montuori C, Settimi S, et al (2022) Efficacy of Biologics on Refractory Eosinophilic Otitis Media Associated with Bronchial Asthma or Severe Uncontrolled CRSwNP. J Clin Med 11:. https://doi.org/10.3390/jcm11040926
6. Mümmler C, Dünzelmann K, Kneidinger N, et al (2021) Real-life effectiveness of biological therapies on symptoms in severe asthma with comorbid CRSwNP. Clin Transl Allergy 11:e12049. https://doi.org/10.1002/clt2.12049
7. Dharmarajan H, Falade O, Lee SE, Wang EW (2022) Outcomes of dupilumab treatment versus endoscopic sinus surgery for chronic rhinosinusitis with nasal polyps. Int Forum Allergy Rhinol 12:986–995. https://doi.org/10.1002/alr.22951
8. Hopkins C, Buchheit KM, Heffler E, et al (2022) Improvement in Health-Related Quality of Life with Dupilumab in Patients with Moderate-to-Severe Asthma with Comorbid Chronic Rhinosinusitis with/without Nasal Polyps: An Analysis of the QUEST Study. J Asthma Allergy 15:767–773. https://doi.org/10.2147/JAA.S363527
9. Ryser FS, Yalamanoglu A, Valaperti A, et al (2023) Dupilumab-induced eosinophilia in patients with diffuse type 2 chronic rhinosinusitis. Allergy 78:2712–2723. https://doi.org/10.1111/all.15844
10. Bertlich M, Ihler F, Bertlich I, et al (2021) Management of chronic rhinosinusitis with nasal polyps in Samter triad by low-dose ASA desensitization or dupilumab. Medicine (Baltimore) 100:e27471. https://doi.org/10.1097/MD.0000000000027471
11. Pecorari G, Piazza F, Borgione M, et al (2023) The role of intranasal corticosteroids in chronic rhinosinusitis with nasal polyposis treated with dupilumab. Am J Otolaryngol 44:103927. https://doi.org/10.1016/j.amjoto.2023.103927
12. Bajpai S, Marino MJ, Rank MA, et al (2021) Benefits of biologic therapy administered for asthma on co-existent chronic rhinosinusitis: A real-world study. Int Forum Allergy Rhinol 11:1152–1161. https://doi.org/10.1002/alr.22774
13. Soyka MB, Ryser FS, Brühlmann C, et al (2023) Predicting dupilumab treatment outcome in patients with primary diffuse type 2 chronic rhinosinusitis. Allergy 78:1036–1046. https://doi.org/10.1111/all.15532
14. Rosso C., De Corso E., Conti V., et al (2024) Switching of biological therapy to dupilumab in comorbid patients with severe asthma and CRSwNP. Eur Arch Oto-Rhino-Laryngol 281:3017–3023. https://doi.org/10.1007/s00405-024-08461-y
15. Ferri S, Montagna C, Casini M, et al (2023) Sleep quality burden in chronic rhinosinusitis with nasal polyps and its modulation by dupilumab. Ann Allergy Asthma Immunol S1081-1206(23)00631–2. https://doi.org/10.1016/j.anai.2023.08.594

*PATIENTS ALREADY INCLUDED IN OTHER STUDY*

1. Ottaviano G, De Corso E, Saccardo T, et al (2023) Effectiveness of Dupilumab in the Treatment of Adult and Older Adult Patients with Severe, Uncontrolled CRSwNP. JPM 13:1241. https://doi.org/10.3390/jpm13081241
2. De Corso E, Settimi S, Montuori C, et al (2022) Effectiveness of Dupilumab in the Treatment of Patients with Severe Uncontrolled CRSwNP: A “Real-Life” Observational Study in the First Year of Treatment. J Clin Med 11:2684. https://doi.org/10.3390/jcm11102684
3. Torretta S, De Corso E, Nava N, et al (2022) Proposal for a Structured Outpatient Clinic for Dupilumab Treatment in Chronic Rhinosinusitis with Nasal Polyps in the First Year of Treatment. J Pers Med 12:1734. https://doi.org/10.3390/jpm12101734
4. Ottaviano G, Saccardo T, Roccuzzo G, et al (2022) Effectiveness of Dupilumab in the Treatment of Patients with Uncontrolled Severe CRSwNP: A “Real-Life” Observational Study in Naïve and Post-Surgical Patients. JPM 12:1526. https://doi.org/10.3390/jpm12091526
5. Galletti C, Barbieri MA, Ciodaro F, et al (2023) Effectiveness and Safety Profile of Dupilumab in Chronic Rhinosinusitis with Nasal Polyps: Real-Life Data in Tertiary Care. Pharmaceuticals (Basel) 16:630. https://doi.org/10.3390/ph16040630
6. van der Lans RJL, Fokkens WJ, Adriaensen GFJPM, et al (2022) Real-life observational cohort verifies high efficacy of dupilumab for chronic rhinosinusitis with nasal polyps. Allergy 77:670–674. https://doi.org/10.1111/all.15134

*INCOMPLETE PUBLISHED OUTCOMES INCLUDING SNOT-22/NPS*

1. Bellocchi G, Loperfido A, Passali FM, et al (2023) Biologics in severe uncontrolled chronic rhinosinusitis with nasal polyps: A bicentric experience. Acta Biomed 94:e2023227. https://doi.org/10.23750/abm.v94i5.14745
2. Al-Ahmad M, Ali A, Khalaf M, et al (2023) Comorbid asthma in patients with chronic rhinosinusitis with nasal polyps: did dupilumab make a difference? BMC Pulm Med 23:266. https://doi.org/10.1186/s12890-023-02556-8
3. Danisman Z, Linxweiler M, Kühn JP, et al (2023) Differential nasal swab cytology represents a valuable tool for therapy monitoring but not prediction of therapy response in chronic rhinosinusitis with nasal polyps treated with Dupilumab. Front Immunol 14:1127576. https://doi.org/10.3389/fimmu.2023.1127576
4. Nettis E, Brussino L, Patella V, et al (2022) Effectiveness and safety of dupilumab in patients with chronic rhinosinusitis with nasal polyps and associated comorbidities: a multicentric prospective study in real life. Clin Mol Allergy 20:6. https://doi.org/10.1186/s12948-022-00171-2
5. Minagawa S, Araya J, Watanabe N, et al (2022) Real-life effectiveness of dupilumab in patients with mild to moderate bronchial asthma comorbid with CRSwNP. BMC Pulm Med 22:258. https://doi.org/10.1186/s12890-022-02046-3
6. Garvey E, Naimi B, Duffy A, et al (2024) Optimizing the timing of biologic and surgical therapy for patients with refractory chronic rhinosinusitis with nasal polyposis (CRSwNP). Int Forum Allergy Rhinol 14:651–659. https://doi.org/10.1002/alr.23246
7. Suzaki I., Maruyama Y., Kamimura S., et al (2024) Residual nasal polyp tissue following dupilumab therapy is associated with periostin-associated fibrosis. Eur Arch Oto-Rhino-Laryngol 281:1807–1817. https://doi.org/10.1007/s00405-023-08336-8
8. Haxel BR, Hummel T, Fruth K, et al (2022) Real-world-effectiveness of biological treatment for severe chronic rhinosinusitis with nasal polyps. Rhin 6:435–443. https://doi.org/10.4193/Rhin22.129
9. Ottaviano G, De Corso E, Cantone E, et al (2023) Measuring Nasal Patency and the Sense of Smell in CRSwNP Patients Treated with Dupilumab. J Pers Med 13:234. https://doi.org/10.3390/jpm13020234
10. Schmale IL, Poulakis A, Abend A, et al (2023) Chronic Rhinosinusitis With Nasal Polyposis Treated With Dupilumab: Real-World Use and Outcomes. J Allergy Clin Immunol Pract 11:3203–3210. https://doi.org/10.1016/j.jaip.2023.07.038
11. Nowsheen S, Darveaux JI (2021) Dupilumab in the treatment of nasal polyposis: A retrospective, real-world study. Ann Allergy Asthma Immunol 127:386–387. https://doi.org/10.1016/j.anai.2021.05.018
12. Riva G, Garetto M, Borgione M, et al (2024) Dupilumab improves sleep quality in chronic rhinosinusitis with nasal polyps. Am J Otolaryngol 45:104310. https://doi.org/10.1016/j.amjoto.2024.104310
13. Ottaviano G, Roccuzzo G, Lora L, et al (2024) The Impact of Dupilumab on Work Productivity and Emotional Health in CRSwNP: A Multicentric Study in Northeast Italy. J Pers Med 14:468. https://doi.org/10.3390/jpm14050468

*LANGUAGE DIFFERENT FROM ENGLISH, SPANISH, PORTUGUESE OR ITALIAN.*

1. Boiko NV, Stagnieva IV, Lodochkina OE, Kurbatova NV (2023) [Experience with dupilumab in the treatment of chronic rhinosinusitis with nasal polyps]. Vestn Otorinolaringol 88:46–53. https://doi.org/10.17116/otorino20228804146
2. Larin RA, Mokeeva PP, Grishin AS (2023) [Experience of biological therapy in severe forms of chronic rhinosinusitis with nasal polyps in the conditions of regional healthcare]. Vestn Otorinolaringol 88:51–58. https://doi.org/10.17116/otorino20228802151
3. Knizek Z. (2023) efficacy of dupilumab in patients with chronic rhinosinusitis with nasal polyps with comorbid allergic rhinitis. Alergie 2023:187–191
4. Appel HM, Lochbaum R, Hoffmann TK, Hahn J (2024) [Chronic rhinosinusitis with nasal polyps-extension of dupilumab treatment intervals]. HNO. https://doi.org/10.1007/s00106-024-01487-y
5. Staufenberg A-R, Frankenberger HK, Förster-Ruhrmann U, et al (2024) [Biologic therapy in patients with severe NSAID-exacerbated respiratory disease and previous aspirin desensitization : Results of a multicentric study]. HNO. https://doi.org/10.1007/s00106-024-01433-y

*CONFERENCE/CONGRESS PUBLISHED ABSTRACTS*

1. Bachert C., Mannent L., Naclerio R.M., et al (2015) Dupilumab in chronic sinusitis with nasal polyposis, with and without asthma. Allergy Eur J Allergy Clin Immunol 70:107. https://doi.org/10.1111/all.12715
2. Boscke R., Bruchhage K.L. (2021) Real-life data on the effectiveness and safety of dupilumab in adult patients with uncontrolled Chronic Rhinosinusitis with nasal Polyps (CRSwNP). Laryngo- Rhino- Otol 100:S278–S279. https://doi.org/10.1055/s-0041-1728626
3. Cipolla F., La Mantia I. (2023) Chronic rinosinusitis with nasal polyps and Dupilumab: Results after six months of treatment. Allergy Eur J Allergy Clin Immunol 78:421–422. https://doi.org/10.1111/all.15616
4. De Prado Gomez L., Khan A.H., Peters A., et al (2023) EValuating trEatment RESponses of dupilumab versus omalizumab in type 2 patients: The EVEREST trial. Allergy Eur J Allergy Clin Immunol 78:616–617. https://doi.org/10.1111/all.15616
5. Dennis S.K., Martin L., Marc D., et al (2022) CRSwNP and Dupilumab-Experiences after one year of clinical use at a university ENT clinic. Laryngo- Rhino- Otol 101:S329. https://doi.org/10.1055/s-0042-1747025
6. Mathias H., Robert B., Marius T., et al (2022) Dupilumab-treatment for uncontrolled CRSwNP: real-life 15-months follow-up data. Laryngo- Rhino- Otol 101:S332. https://doi.org/10.1055/s-0042-1747000
7. Schmale I., Poulakis A., Abend A., et al (2023) Real-world Outcomes of Dupilumab Therapy for Nasal Polyposis in Patients with Aspirin-Exacerbated Respiratory Disease. J Allergy Clin Immunol 151:AB218. https://doi.org/10.1016/j.jaci.2022.12.680
8. Hoffmann A.S., Eden J., Jansen F., et al (2021) Efficacy of Dupilumab (Dupixent) in the treatment of chronic rhinosinusitis with nasal polyps: First results from Hamburg. Laryngo- Rhino- Otol 100:S284–S285. https://doi.org/10.1055/s-0041-1728858
9. Sophie H.A., Florian J., Benjamin B., et al (2022) Real world data on the effectiveness and safety of dupilumab in adult patients with uncontrolled severe chronic rhinosinusitis with nasal polyps (CRSwNP). Laryngo- Rhino- Otol 101:S328. https://doi.org/10.1055/s-0042-1747020
10. Ferreira Gom M.P., Ferreira S., Gomes Rosa J.P., et al (2024) Chronic Rhinosinusitis With Nasal Polyposis: Portuguese Experience With Dupilumab In Two Centers. J Allergy Clin Immunol 153:AB214. https://doi.org/10.1016/j.jaci.2023.11.691
11. Juarez C., Eusebio I., Viciana M.G., et al (2024) Dupilumab Effectivity in Chronic Rhinosinusitis with Nasal Polyps and Asthma in Real-Life Conditions. J Allergy Clin Immunol 153:AB103. https://doi.org/10.1016/j.jaci.2023.11.337
12. Lanz M., Eisenlohr C., Chartrand E., Herrera L. (2024) Early Clinical Improvement Of Anosmia And Sinus Nitric Oxide In CRSwNP Subjects Treated With Dupilumab. J Allergy Clin Immunol 153:AB210. https://doi.org/10.1016/j.jaci.2023.11.677
13. Peters A., Han J., Goeckner B., et al (2024) Efficacy of Biologics in Patients With Moderate-to-Severe, Uncontrolled Asthma and Nasal Polyps. J Allergy Clin Immunol 153:AB100. https://doi.org/10.1016/j.jaci.2023.11.329
